# Comparative genomic analysis of *Klebsiella pneumonia* (LCT-KP214) and a mutant strain (LCT-KP289) obtained after spaceflight

**DOI:** 10.1186/1471-2164-15-589

**Published:** 2014-07-12

**Authors:** Yinghua Guo, Yinhu Li, Longxiang Su, De Chang, Wenbin Liu, Tong Wang, Yanting Yuan, Xiangqun Fang, Junfeng Wang, Tianzhi Li, Chengxiang Fang, Wenkui Dai, Changting Liu

**Affiliations:** 1 Nanlou Respiratory Diseases Department, Chinese PLA General Hospital, Beijing 100853, China; 2 BGI-Shenzhen, Shenzhen 518083, China; 3 Department of Critical Care Medicine, Peking Union Medical College Hospital, Peking Union Medical College & Chinese Academy of Medical Sciences, Beijing 100730, China; 4 College of Life Sciences, Wuhan University, Wuhan 430072, China; 5 Department of Respiratory Medicine, General Hospital of Chinese People's Armed Police Forces, Beijing 100039, China

**Keywords:** *Klebsiella pneumoniae*, Comparative genomic analysis, Virulence gene, Resistance gene

## Abstract

**Background:**

With the development of space science, it is important to analyze the relationship between the space environment and genome variations that might cause phenotypic changes in microbes. *Klebsiella pneumoniae* is commonly found on the human body and is resistant to multiple drugs. To study space-environment-induced genome variations and drug resistance changes, *K. pneumoniae* was carried into outer space by the Shenzhou VIII spacecraft.

**Results:**

The *K. pneumoniae* strain LCT-KP289 was selected after spaceflight based on its phenotypic differences compared to the ground-control strain. Analysis of genomic structural variations revealed one inversion, 25 deletions, fifty-nine insertions, two translocations and six translocations with inversions. In addition, 155 and 400 unique genes were observed in LCT-KP214 and LCT-KP289, respectively, including the gene encoding dihydroxyacetone kinase, which generates the ATP and NADH required for microbial growth. Furthermore, a large number of mutant genes were related to transport and metabolism. Phylogenetic analysis revealed that most genes in these two strains had a dN/dS value greater than 1, indicating that the strain diversity increased after spaceflight. Analysis of drug-resistance phenotypes revealed that the *K. pneumoniae* strain LCT-KP289 was resistant to sulfamethoxazole, whereas the control strain, LCT-KP214, was not; both strains were resistant to benzylpenicillin, ampicillin, lincomycin, vancomycin, chloramphenicol and streptomycin. The sulfamethoxazole resistance may be associated with sequences in Scaffold7 in LCT-KP289, which were not observed in LCT-K214; this scaffold contained the gene *sul1*. In the strain LCT-KP289, we also observed a drug-resistance integron containing *emrE* (confers multidrug resistance) and *ant* (confers resistance to spectinomycin, streptomycin, tobramycin, kanamycin, sisomicin, dibekacin, and gentamicin). The gene *ampC* (confers resistance to penicillin, cephalosporin-ii and cephalosporin-i) was present near the integron. In addition, 30 and 26 drug-resistance genes were observed in LCT-KP289 and LCT-KP214, respectively.

**Conclusions:**

Comparison of a *K. pneumoniae* strain obtained after spaceflight with the ground-control strain revealed genome variations and phenotypic changes and elucidated the genomic basis of the acquired drug resistance. These data pave the way for future studies on the effects of spaceflight.

## Background

With the rapid development of space technology, space capsules are frequently launched to explore the universe; therefore, it is important to understand space biology. Space environmental physics has elucidated the existence of properties including electromagnetic radiation, microgravity, high vacuum and strong magnetic field in the space environment; however, it is important to understand the impact of these factors on organisms. Paul *et al.* focused on plants, which are significant components of biological systems, and discussed the adaption and growth tropism of plants in the microgravity environment in a space shuttle [1]. Gridhani *et al.* examined proton-induced perturbations in gene expression, cell cycle and cell division as well as the differences between the effects of protons and high-energy proton radiation [2,3]. Gao *et al.* observed that bacterial metabolism was significantly altered in the space environment [4]; furthermore, exposure to the space environment might cause genetic damage [5]. Tixador *et al.* studied the growth and antibiotic resistance of *Escherichia coli* during the mission of the space shuttle Discovery [6]. However, mutations caused by the space environment have not been examined at the genomic level. *Klebsiella pneumoniae* is an important Gram-negative, opportunistic pathogen that causes severe diseases such as septicemia, pneumonia, urinary tract infections, and soft-tissue infections [7]. Many clinical strains of *K. pneumoniae* are highly resistant to antibiotics, which poses a major threat to global public health. Over the past decade, the physiology, biochemistry, and regulation of *K. pneumoniae* pathways have been extensively studied [8-11]. However, the effect of spaceflight on *K. pneumoniae* has not been examined at the genomic level. *K. pneumoniae* is well-suited for such studies because of its characteristics.

In 2011, the Shenzhou VIII spacecraft carried *K. pneumoniae* strains into outer space for approximately 17 days (398 hours). The control strain was cultured at the same temperature in an incubator on earth. After spaceflight, the antibiotic resistance and pathogenicity of the strains were examined. Based on these analyses, the LCT-KP289 strain obtained after spaceflight was selected and compared to the control strain LCT-KP214. The genomes of LCT-KP289 and LCT-KP214 were sequenced to compare their genomic variations. These analyses revealed genes potentially related to drug resistance, and analysis of the putative drug-resistance genes revealed variations in the homologous genes in the two strains. Studies on these candidate resistance genes will be important to improve understanding of the drug resistance of *K. pneumoniae*.

## Results

### Genomic features of the strains LCT-KP214 and LCT-KP289

The genome sequences of LCT-KP214 and LCT-KP289 were deposited in NCBI under the GenBank accession numbers AJHE00000000 and ATRO00000000, respectively. The genomes are described in Table 1. Genome alignments revealed that most regions were present in both strains, including 339 alignment blocks that covered 95.90% of the LCT-KP214 genome and 91.45% of the LCT-KP289 genome. The strain LCT-KP289 exhibited a larger genome size, and the extra sequences mainly comprised repetitive sequences (including tandem repeat fragments and interspersed repeated sequences).

**Table 1:** Sequence assembly data for LCT-KP214 and LCT-KP289

**Strain name**	**LCT-KP214**	**LCT-KP289**
**Genome size**	5,791,462 bp	6,068,157 bp
**GC content**	56.64%	84.42%
**Gene number**	5,907	6,155
**Average gene length**	828 bp	816 bp
**Genome coding percentage**	84.42%	82.87%
**TRF**	17,084 bp	62,013 bp

**Figure 1:**
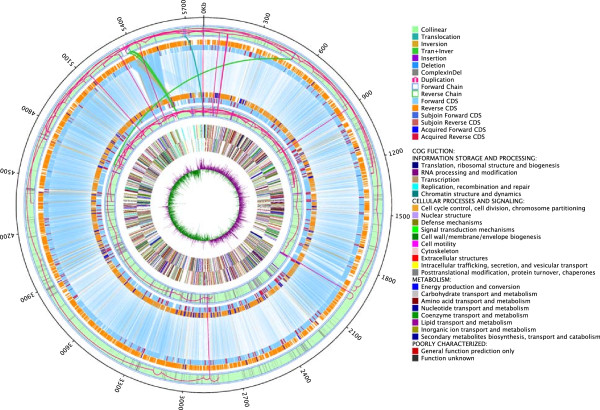
**Genomic structural variation and distribution of paired genes.** The structural variations in the genomes and paired genes are shown. The circles represent (inner to outer) the LCT-KP214 GC-skew distribution, LCT-KP214 COG distribution, and the structural variations between LCT-KP214 and LCT-KP289, respectively. LCT-KP214 GC-skew distribution: a 1,000-bp sliding window was used on the genome of LCT-KP214, and the GC-skew was recorded in each window. GC-skew = (G-C)/(G + C); purple and green indicate positive and negative values, respectively. The junction between purple and green represents the potential transcription initiation site. LCT-KP214 COG distribution: the inner, second and third circles represent the COG functional distribution in LCT-KP214. Different functions are indicated by different colors, and the captions are listed on the right. Structural variations between LCT-KP214 and LCT-KP289: the circles indicate the genomic structural variation, syntenic regions and gene pairs in LCT-KP214 and LCT-KP289 (the genes located on the forward strand are indicated in sky blue, and the genes located on the reverse strand are indicated in orange). The outer circles represent LCT-KP214, and the inner circles represent LCT-KP289. The lines connecting the circles indicate paired genes in LCT-KP214 and LCT-KP289, and the different colors indicate structural variations, transfer elements and duplicated sequences.

Based on the assembly results, the genes in strains LCT-KP214 and LCT-KP289 were predicted and annotated using COGs (Cluster of Orthologous Groups). Using this analysis, 3,791 and 3,935 genes were assigned to specific gene functions in strains LCT-KP214 and LCT-KP289, respectively (Figure 1). Genome alignment revealed that most genes in LCT-KP289 were present in LCT-KP214 and that the additional genes in LCT-KP289 were most likely repeated sequences. The unique genes in these strains are discussed below. The predicted genes were annotated using the ARDB (Antibiotic Resistance Genes Database), and 26 and 30 genes were assigned antibiotic-resistance functions in LCT-KP214 and LCT-KP289, respectively. The assembly results were then annotated using plasmid databases, and some scaffolds in LCT-KP214 and LCT-KP289 were homologous to plasmid genomes. The plasmid annotation analysis revealed that some plasmids did not belong to *K. pneumoniae* (Table 2). However, the presence and origin of these plasmids require further analysis.

**Table 2:** Statistics of plasmid alignment results in the two strains

**Strain Name**	**Scaffold**	**Length (bp)**	**Coverage (%)**	**Plasmid ID**	**Length (bp)**	**Coverage (%)**	**Annotation**
LCT-KP214	Scaffold2	192,758	74.85	NC_019390.1	207,819	70.06	*Klebsiella pneumoniae* plasmid pKPN_CZ
LCT-KP214	Scaffold4	90,588	84.83	NC_009133.1	94,289	80.17	*Escherichia coli* plasmid NR1
LCT-KP214	Scaffold3	165,023	75.04	NC_019158.1	137,813	87.54	*Klebsiella pneumoniae* plasmid pNDM10469
LCT-KP289	Scaffold2	193,508	74.70	NC_019390.1	207,819	70.14	*Klebsiella pneumoniae* plasmid pKPN_CZ
LCT-KP289	Scaffold3	164,884	75.65	NC_019153.1	162,746	74.62	*Klebsiella pneumoniae* plasmid pNDM-KN
LCT-KP289	Scaffold4	98,792	82.67	NC_009133.1	94,289	80.26	*Escherichia coli* plasmid NR1
LCT-KP289	Scaffold7	2,242	100.00	NC_015599.1	54,205	4.14	*Escherichia coli* IncN plasmid N3
LCT-KP289	Scaffold8	1,359	96.17	NC_004604.2	53,865	2.43	*Bacillus megaterium* QM B1551 plasmid pBM400
LCT-KP289	Scaffold9	1,154	99.13	NC_016009.1	19,557	5.85	*Enterococcus faecium* plasmid pM7M2
LCT-KP289	Scaffold10	950	98.53	NC_013317.1	21,806	4.29	*Enterococcus faecium* plasmid p5753cA

### Detection of genomic structural variations and functional enrichment of variant genes

**Figure 2:**
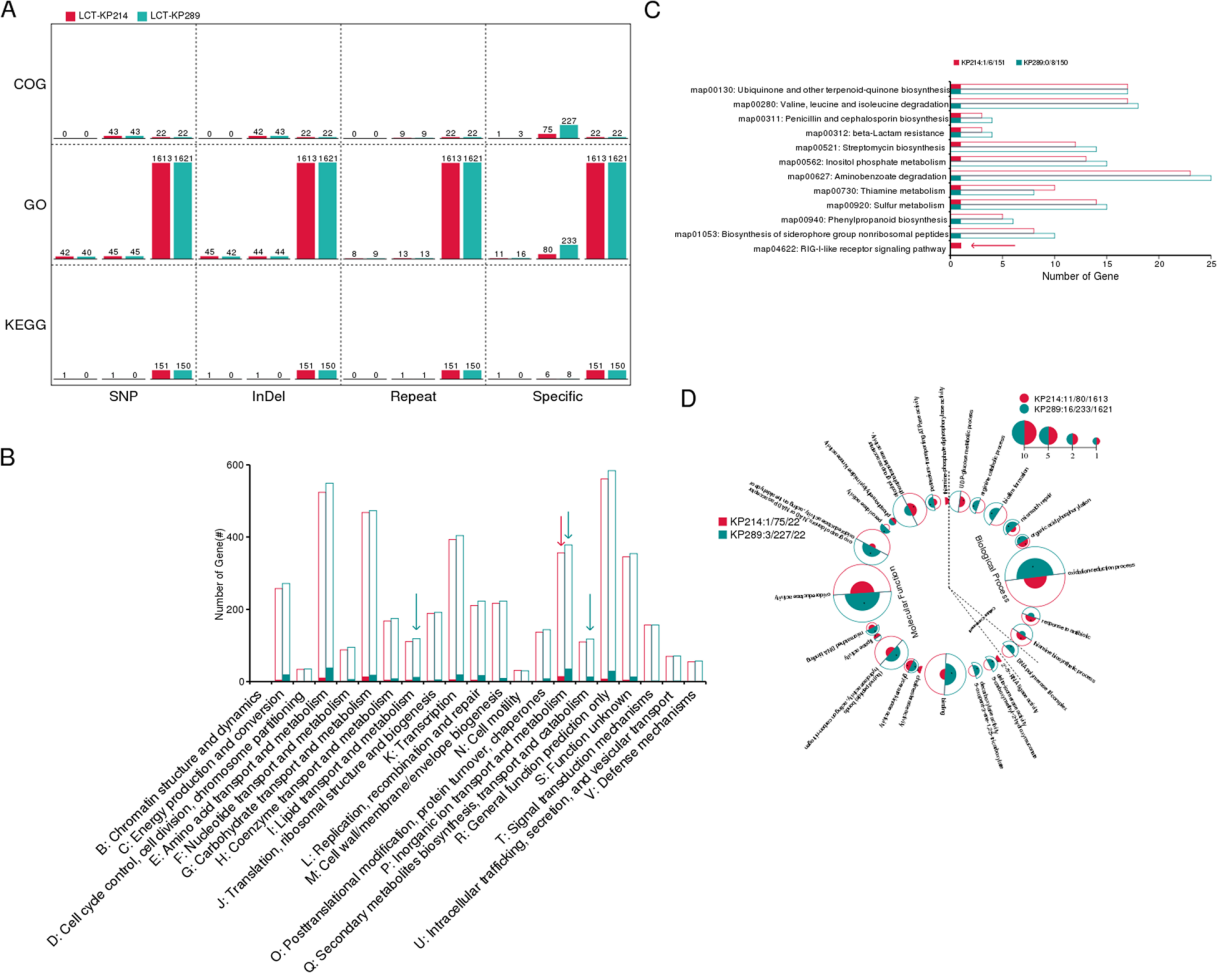
**Functional enrichment of variant genes after KEGG/COG/GO annotation.** Picture **A**: Analysis of functional enrichment. The red and green bars represent LCT-KP214 and LCT-KP289, respectively. The genes containing SNPs or InDels, those located in repeat regions or those unique to each strain were analyzed separately. Each classification square contained three paired bars, indicating the number of enriched functional items, the number of annotated variant genes, and the number of all annotated genes in the strain from left to right. Pictures **B**, **C** and **D** indicate the detailed enrichment results of variant genes after COG, KEGG and GO annotation. In pictures **B**, **C**, and **D**, the annotation axis is classified by functional items. The hollow columns represent the number of all annotated genes in the strain. The solid columns represent the number of annotated variant genes. The arrows in pictures **B** and **C** and the asterisk in picture **D** indicate significantly enriched functional items.

The genomic variations in LCT-KP214 and LCT-KP289 were analyzed and the genomic differences, including sequence variants, were identified (Figure 1). We defined variant genes as those that contained SNPs or InDels, were partly located in syntenic or repeat regions, or were unpaired (genes present at similar loci in both strains were considered paired). Subsequently, the variant genes in strains LCT-KP214 and LCT-KP289 were annotated using the COG/GO/KEGG databases and classified by their functions (Figure 2A).Analysis of genome variation revealed 1 inversion, 25 deletions, 59 insertions, 1 translocation and 6 translocations with inversions (Figure 1). Genes outside syntenic regions (mainly separated by large insertions or deletions) were identified, including 389 and 629 genes, respectively, in LCT-KP214 and LCT-KP289. In addition, the two strains contained some homologous genes. After filtering the homologous genes, we observed 155 and 400 unique genes in LCT-KP214 and LCT-KP289, respectively. Based on COG annotations, the functional categories “Lipid transport and metabolism”, “Inorganic ion transport and metabolism” and “Secondary metabolites biosynthesis, transport and catabolism” were significantly enriched in the unique genes in LCT-KP289; the functional category “Inorganic ion transport and metabolism” was significantly enriched in the unique genes in LCT-KP214 (Figure 2B). Furthermore, we detected 11 interspersed repeated sequences with duplication units longer than 400 bp, and the copy numbers of the repeated sequences were different. The genes located in the repeat regions were not significantly enriched in COG or KEGG functional categories; however, 9 gene functional categories were enriched based on GO annotations, including “fatty acid metabolic process”, “peptidoglycan biosynthetic process”, and “peptidoglycan-based cell wall” (Figure 2D).

In addition to the large-scale genome variations, 770 SNPs were identified (3,133 raw SNPs; 2,363 SNPs containing N bases, nearly N bases or with low sequencing depth were filtered). To analyze the genes affected by SNPs, we selected SNPs located in CDS or intergenic regions no further than 300 bp from the nearest gene. We observed that 334 and 330 genes were affected by SNPs in LCT-KP289 and LCT-KP214, respectively. Furthermore, 99 InDels were also identified (133 raw InDels; 34 InDels containing Ns or with low sequencing depth were filtered). InDel-affected genes were identified using the method described for SNPs; 299 and 304 genes were affected by InDels in LCT-KP289 and LCT-KP214, respectively (not all InDels were located in CDS regions in both strains). Based on the GO annotations, 48 and 49 functional categories were enriched in the genes containing SNPs and InDels, respectively, in the strain LCT-KP289. Most of the affected genes encoded molecular functions related to transmembrane transporters (Additional file 1: Table S1). Based on KEGG annotations, the unique gene KP214_00701 in LCT-KP214 was assigned the function “Glycerolipid metabolism (ko00561)” [Figure 2C]; this gene contained 12 SNPs and 5 InDels. KP214_00701 encodes dihydroxyacetone kinase (*dha*), which is required to generate ATP and NADH for microbial growth [12]. However, this gene was not functional in LCT-KP289 because of a nonsense mutation (GAA- > TAA). This mutation in LCT-KP289 might have been caused by the severe environmental changes during spaceflight.

### Analysis of phylogenetic relationships

**Figure 3:**
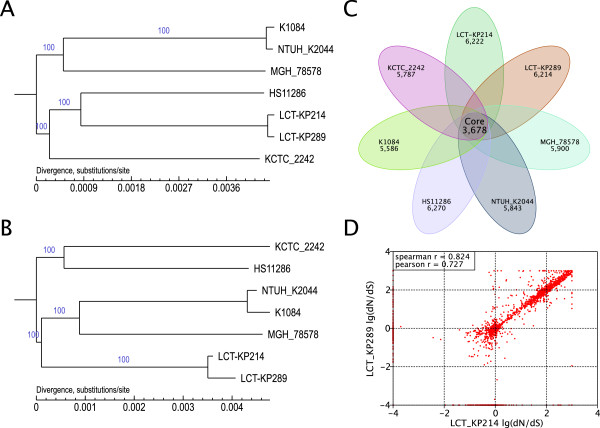
**Phylogenetic analysis.** Picture **A**: A phylogenetic tree was constructed at the genome level, and the phylogenetic relationships among these 7 strains are shown. Picture **B**: A phylogenetic tree was constructed at the gene level. The base substitution rates of the genes in LCT-KP289 were higher compared to those in LCT-KP214. Picture **C**: The distribution of the number of genes in each *K. pneumoniae* strain and the number of their core genes. Picture **D**: dN/dS comparison between LCT-KP214 and LCT-KP289. For the paired samples, the p value was 0.001989 by the Wilcoxon test and 2.66e-06 by Student's *t*-test.

To understand the effect of the environment on strain evolution, the phylogenetic relationship between LCT-KP214 and LCT-KP289 was analyzed, and the base substitution rates were compared. We downloaded five other *K. pneumoniae* genome sequences from NCBI (ftp://ftp.ncbi.nih.gov/genbank/genomes/Bacteria) and used Bayesian analysis to generate phylogenetic trees at the whole-genome (Figure 3A) and gene levels (Figure 3B). The base substitution rates in LCT-KP214 and LCT-KP289 were 3.4e-05 and 4.6e-05, respectively, at the genome level, and 5e-06 and 5.7e-04, respectively, at the gene level. Therefore, LCT-KP289 had higher base substitution rates at the genome and gene levels. We used the phylogenetic trees and 3,678 core genes of the seven *K. pneumoniae* strains (Figure 3C) to calculate the dN/dS ratios in LCT-KP214 and LCT-KP289 using the CODONML software (in PAML version 4.4, January 2010) with the GY-HKY model. Comparison of LCT-KP214 and LCT-KP289 revealed that most genes in these strains shared the same selection pressure, and the dN/dS value of most of the genes was greater than 1 (Figure 3D), which indicated that the strains tended to have greater diversity under special circumstances, such as spaceflight.

**Figure 4:**
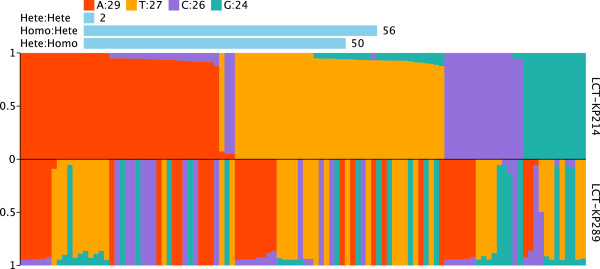
**Base frequencies of the core genomes of LCT-KP214 and LCT-KP289.** Homo indicates that the base pair was homozygous at the locus, and hete indicates that the base pair was heterozygous at the locus. Homo:Hete indicates that the base-types were stable in LCT-KP214, but not in LCT-KP289; Hete:Homo indicates that the base-types were stable in LCT-KP289, but not in LCT-KP214; Hete:Hete indicates that the base-types were not stable in LCT-KP214 or LCT-KP289.

Furthermore, heterozygotic SNPs, which might reflect differential rates of evolution, were observed in these two strains. We mapped the reads to the assembly results and selected bases with a query value greater than 20 to analyze SNP heterozygosis. We selected heterozygotic SNPs that satisfied the following criteria: frequency of the second-most abundant nucleotide greater than 0.05 and total depth greater than 50X. The comparison of the base frequencies in the core genomes of strains LCT-KP214 and LCT-KP289 is shown in Figure 4. We observed that most base types were stable in LCT-KP214, but strain LCT-KP289 had increased base-type diversity. The statistics of the annotation results in Table 3 indicated that most heterozygotic SNPs were nonsynonymous. The analysis also revealed that the *K. pneumoniae* bacterial colonies had greater genome variation after spaceflight.

**Table 3:** Statistics of the annotation results for heterozygotic SNPs

**Mutation Type**	**LCT-KP214**		**LCT-KP289**	
	**Number**	**Percentage (%)**	**Number**	**Percentage (%)**
Start codon nonsyn	0	0.00	0	0.00
Stop codon nonsyn	0	0.00	1	1.43
Premature stop	0	0.00	0	0.00
Nonsynonymous	41	73.21	44	62.86
Start codon syn	0	0.00	0	0.00
Stop codon syn	0	0.00	0	0.00
Synonymous	9	16.07	11	15.71
Total Mutated	50	89.29	56	80.00
SNP within CDS	49	87.50	56	80.00
Intergenic SNP	7	12.50	14	20.00
Total SNP	56	100.00	70	100.00

### Characterization of drug-resistance and related genes

**Figure 5:**
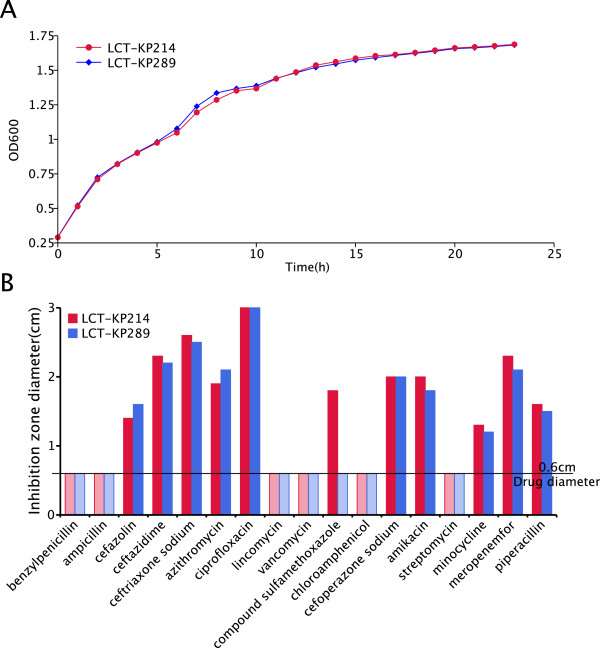
**Phenotypic analyses.** Picture **A**: The growth curves of LCT-KP214 and LCT-KP289 were determined by measuring OD600 values, which represent the concentration of the bacterial population. The growth curves of LCT-KP214 and LCT-KP289 were nearly identical. Picture **B**: The antibiotic resistance phenotypes were characterized by diffusion tests. The diameter of the zone of inhibition was measured for each antibiotic, and the drug was applied in a 0.6-cm diameter circle. If the diameter of the zone of inhibition of an antibiotic was 0.6 cm after incubation of the bacterial plate, the strain was considered resistant to the antibiotic. The data showed that LCT-KP289 was resistant to sulfamethoxazole, but LCT-KP214 was not.

Spaceflight can cause genome variation that leads to alterations in bacterial drug resistance [13,14]. We measured the growth rates and characterized the drug resistance phenotypes of strain LCT-KP289 after spaceflight and of the ground-control strain LCT-KP214. Both strains had similar growth curves (Figure 5A). However, the two strains differed in their resistance to sulfamethoxazole; LCT-KP289 was resistant to this compound, but LCT-KP214 was not. Both strains exhibited similar resistance to benzylpenicillin, ampicillin, lincomycin, vancomycin, chloramphenicol, and streptomycin (Figure 5B).

**Figure 6:**
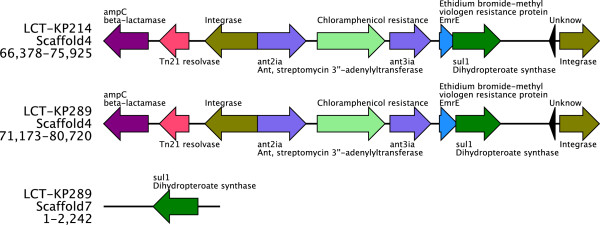
**Gene elements near the *sul1 g*ene.** Two copies of the *sul1* gene were observed in LCT-KP289, but only one copy was observed in LCT-KP214. We observed two integrases in the vicinity of the *sul1* gene in LCT-KP214 and LCT-KP289. Between these genes, other resistance genes, such as *ant*, which confers resistance to spectinomycin, streptomycin, tobramycin, kanamycin, sisomicin, dibekacin and gentamicin, were observed. The gene *ampC*, which confers resistance to penicillin, cephalosporin-ii and cephalosporin-I, was also observed near the *sul1* gene. The different-colored arrows indicate the direction and length of the genes.

The sulfonamide sulfamethoxazole might inhibit dihydrofolate synthetase and repress DNA synthesis in *K. pneumoniae*. To identify the sulfamethoxazole-resistance mechanism in LCT-KP289, we analyzed the ARDB annotation results and observed that the *sul1* gene, which is important for sulfonamide resistance, was duplicated. LCT-KP214 had only one copy of *sul1* (in Scaffold4), but LCT-KP289 had two copies of this gene (in Scaffold4 and Scaffold7). *sul1* encodes an alternative dihydrofolate synthetase that is not inhibited by sulfonamide; therefore, sulfonamides cannot block the synthesis of dihydropteroate, leading to sulfonamide resistance [15]. Plasmid annotation analysis revealed that the scaffolds containing the *sul1 g*ene were homologous to plasmid genomes (Table 2). As the *sul1* gene is mostly associated with class 1 integrons, we decided to explore the genetic context of *sul1*. In both strains, the *sul1* gene was part of a class 1 integron (Figure 6). Integrons can capture gene cassettes by site-specific recombination and drive the expression of these genes. Integrons are present in plasmids or transposons [16], which leads to the spread of drug-resistance genes. In addition to the integrase (*intI*) gene, Class 1 integrons usually contain genes encoding resistance to quaternary ammonium compounds, ethidium bromide (*qacED*) and sulfonamides (*sul1*) [17-19]. In this study, LCT-KP214 and LCT-KP289 contained resistance integrons (RI), which included the gene *emrE* (encoding the ethidium bromide-methyl viologen resistance protein) and other resistance genes such as *ant* (ant2ia and ant3ia encode streptomycin 3’-adenylyltransferase and aminoglycoside o-nucleotidyl transferase, respectively, which can modify aminoglycosides such as spectinomycin, streptomycin, tobramycin, kanamycin, sisomicin, dibekacin and gentamicin by adenylation) (Figure 6). In addition to integrons, transposon resolvase (Tn21) and the gene *ampC* were observed near the *sul1* gene. *ampC* encodes a Class A beta-lactamase, which confers resistance to penicillin, cephalosporin-ii and cephalosporin-i. In addition, 30 genes in LCT-KP289 and 26 genes in LCT-KP214 were assigned to ARDB, and these genes are important for *K. pneumoniae* antibiotic resistance (Additional file 2: Table S2).

## Discussion

Based on the assembly results, the genome of LCT-KP289 was larger than that of LCT-KP214; genome alignment and prediction of genome elements revealed that the extra sequences in LCT-KP289 were from tandem repeat fragments and interspersed repeated sequences. Analysis of genome variation revealed that LCT-KP289 contained more unique genes than LCT-KP214. GO functional annotation revealed that the unique genes of LCT-KP289 were enriched in 9 functional items, including oxidation-reduction, biofilm formation, and arginine catabolism. Therefore, the genomic changes in LCT-KP289 were related to environmental adaptation. We analyzed the evolutionary rate and phylogenetic relationships of LCT-KP289. The base frequencies of the core genomes revealed a greater number of heterozygotic SNPs in LCT-KP289 compared to LCT-KP214. The phylogenetic trees at the genome and gene levels indicated that LCT-KP289 had a greater base-substitution rate. The dN/dS analysis revealed that most genes in LCT-KP289 had dN/dS values greater than 1. Together, these data indicated that special environmental factors can stimulate base substitutions in bacteria and that environmental selection pressure might influence the direction of bacterial evolution [20].

To identify the genes that were most affected by the environment, the variant genes in LCT-KP214 and LCT-KP289 were annotated using the COG, KEGG and GO databases. These annotations revealed that some functional categories were enriched in LCT-KP289 compared to LCT-KP214; these categories were related to bacterial metabolism such as “Lipid transport and metabolism”, “Inorganic ion transport and metabolism” and “Secondary metabolites biosynthesis, transport and catabolism”. However, using KEGG annotation, LCT-KP289 lacked the dihydroxyacetone kinase gene, which is important for the generation of ATP and NADH, because of a premature stop codon [21]. Therefore, we propose that, in LCT-KP289, mutations occurred more frequently in genes related to environmental adaptation.

*K. pneumoniae* is highly pathogenic and encodes multiple types of drug resistance genes. In this study, we analyzed the drug-resistance phenotypes of LCT-KP214 and LCT-KP289. Both strains were resistant to several drugs, except sulfamethoxazole, and LCT-KP289 was more resistant to this drug than was LCT-KP214. Based on genome alignment and ARDB annotation, we observed that LCT-KP289 contained an extra copy of the *sul1 g*ene in scaffold7. *sul1* encodes an alternative form of dihydrofolate synthetase, which cannot be inhibited by the drug [22]. Therefore, environmental selection pressure might cause genome variations, leading to an additional copy of the *sul1* gene in LCT-KP289 and subsequent enhanced drug resistance.

Furthermore, we observed gene elements related to antibiotic resistance in *K. pneumoniae.* Integron determinants, including gene cassettes and several antibiotic-resistance genes (e.g., *emrE*, *ant* and s*ul1*), were observed in both strains. Antibiotics can be divided into four classes based on their mechanism of action: i) repression of bacterial cell-wall synthesis, ii) destruction of the cell membrane structure, iii) repression of protein synthesis, and iv) repression of DNA synthesis. *emrE* encodes an efflux pump that confers multidrug resistance [23]. The protein encoded by the gene *ant* potentially modifies aminoglycosides by adenylation, conferring resistance to protein synthesis inhibitors such as spectinomycin, streptomycin, tobramycin, kanamycin, and gentamicin [24]. Because integrons are mobile genetic elements, the antibiotic genes contained in integrons can be transferred among different strains. Based on plasmid annotation, we observed that the integrons in LCT-KP289 and LCT-KP214 might originate from *Escherichia coli* plasmids. We observed the gene *ampC,* which encodes beta-lactamase, flanking the integrons. This enzyme can inactivate cell-wall synthesis inhibitors such aspenicillin and cephalosporin [25]. Furthermore, the Tn21 resolvase was observed near the integrons, which might enhance the mobility of the antibiotic-resistance genes. Other antibiotic-resistance genes, including those related to polymyxin and bacitracin, were dispersed in the genome of *K. pneumoniae.*

## Conclusion

In this study, comparative genomics was used to analyze the strain LCT-KP289, which was selected after spaceflight, and the ground-control strain LCT-KP214 to identify the relationship between the unique space environment and genome variation; furthermore, the potential rate of evolution was studied using phylogenetic analysis. We observed that the space environment affected the evolutionary rates of environment-related genes. Phenotypic analysis revealed that LCT-KP289 had increased resistance to sulfonamides, potentially due to an increased copy number of the gene *sul1*. Furthermore, the observation of integrons in *K. pneumoniae* provides insight into the mechanism of multidrug resistance. The HMP data have shown that the bacteria were multidrug resistant and highly pathogenic. Therefore, this study paves the way for future studies on the effect of spaceflight on drug resistance and pathogenicity.

## Methods

### Bacterial strains and growth conditions

The *K. pneumoniae* strain (CGMCC 1.1736) used in this study was obtained from the Chinese General Microbiological Culture Collection Center and was carried in the Shenzhou VIII unmanned spacecraft for more than 17 days (398 hours). This strain is NCTC 5056, which was clinically isolated from patients diagnosed with pneumonia. The strains were cultured in LB, which contained tryptone (10 g/liter), yeast extract (5 g/liter), NaCl (10 g/liter), and agar powder (15 g/liter). The pH of the culture medium was adjusted to 7.0-7.2. The culture broth was identical to the culture medium but lacked Bacto Agar. The Shenzhou VIII unmanned spacecraft was launched by a Long March 2 F rocket at 5:58 on November 1, 2011 (GMT + 8). It arrived at the Tiangong-1 space station (approximately 340 km apogee distance) and successfully docked with the space station at 17:28(GMT + 8). After docking, the Shenzhou VIII spacecraft and Tiangong-1 remained connected for 12 days at an approximate apogee distance of 350 km. The Shenzhou VIII spacecraft successfully separated from the Tiangong-1 space station and docked again on November 14. Subsequently, the Shenzhou VIII spacecraft remained with the Tiangong-1 space station for three more days. The return capsule of the Shenzhou VIII spacecraft completed the experimental task and left the Tiangong-1 space station on November 17. It landed in the Inner Mongolia region at 20:38 on November 17 (GMT + 8). Microbiological samples were quickly removed and transported to Beijing by military helicopters. The samples arrived at the laboratory of the Chinese PLA General Hospital at 7:17 on November 18 (GMT + 8). As a control, the same *K. pneumoniae* strain was maintained in a laboratory incubator on earth under the same temperature conditions as the cabin of the Shenzhou VIII spacecraft. The temperature conditions were adjusted according to the spacecraft conditions. During spaceflight, the temperature fluctuated between 16-21°C. Because the warehouse was not equipped with a radiation-measuring device, we were unable to obtain relevant data. After the Shenzhou VIII spacecraft landed, the bacterial colonies were randomly selected from plates coated with *K. pneumoniae*. Phenotypic analyses, including disk diffusion tests and growth curves, were performed to compare the spaceflight clones and the ground control strain (Additional file 3: Figure S1). The ground control strain was named LCT-KP214. One clone that was obtained after spaceflight and was significantly different from LCT-KP214 was named LCT-KP289.

### Growth curve and antibiotic-resistance analyses

The strains were grown on LB liquid medium for 18 h at 37°C. Approximately 20-μl suspensions were inoculated into microtiter plates (honeycomb plates) containing 350 μl LB broth and detected by Bioscreen C (Lab Systems, Helsinki, Finland) at 37°C with continuous shaking. The growth of each sample was monitored by measuring the optical density at 600 nm (OD600) at three time points. A well containing 370 μl LB was included as a negative control. The growth curve of each strain was generated based on the OD600 measurements. The *K. pneumoniae* strains were transferred from the plate culture system to 1.5-ml centrifuge tubes containing 1 ml of physiological saline, and the concentrations of the bacterial suspension were diluted to 10^7^ ~ 10^8^ bacteria per ml. Culture plates were then coated with 100 μl of bacterial suspension of each strain. Tablets of filter paper were moistened with 17 different antibiotics, including benzylpenicillin, ampicillin, cefazolin, ceftazidime, ceftriaxone sodium, azithromycin, ciprofloxacin, lincomycin, vancomycin, the pediatric compound sulfamethoxazole, chloramphenicol, cefoperazone sodium, amikacin, streptomycin, minocycline, meropenem, and piperacillin. The tablets were then placed on the surface of the culture plates. Each plate contained three types of antibiotics, and two tablets were used for each antibiotic. The plates were then incubated at 37°C for 18 ~ 24 h, and the diameters of the inhibition zones were measured and recorded. The diameter of the tablets was 6 mm.

### DNA sample preparation and sequencing

*K. pneumoniae* genomes were sequenced using an Illumina Hiseq2000 with a multiplexed protocol. Paired-end reads of 90 bp each were generated from 500-bp and 6-kb random sequencing libraries for the control strain LCT-PK214 and the strain obtained after spaceflight, LCT-PK289. We filtered the raw data in four steps: removing reads with 5 bp of Ns, removing reads with 20 bp of low-quality (≤Q20) bases, removing adapter contamination, and removing duplicate reads. Finally, 100X and 50X filtered paired-end reads were obtained for the 500-bp and 6-kb libraries, respectively. The assembly was performed using the SOAPde novo algorithm [26,27] (http://soap.genomics.org.cn/soapdenovo.html, version: 2.04). Local assembly and gap closure were performed on paired-end reads located in gaps. For highly complex regions, PCR gap closure was performed to obtain sequences without outer gaps. Finally, the SOAPaligner/soap2 software was used for error correction [28,29] (http://soap.genomics.org.cn/soapaligner.html, version 2.21). The reads were mapped to the sequence, mapping information was recoded, and single-base and local proofreading were performed to analyze the assembly results.

### Analysis of genomic components and identification of variations

The sequence of the query strain LCT-PK289 was compared to the reference sequence LCT-PK214 using Mummer [30] (http://mummer.sourceforge.net, version 3.22) and LASTZ [31,32] (http://www.bx.psu.edu/miller_lab/dist/README.lastz-1.02.00, Version: 1.01.50). We used Mummer for chain stander and start site selection, and LASTZ was used for detailed alignment. Next, the syntenic regions and structural variations, including deletions, insertions, inversions and translocations, were identified in the alignment blocks [33]. SNPs were identified by measuring the distances between mismatched sites in syntenic regions. SNPs located in sequence gaps, repeat regions, or scaffold ends were discarded. To validate the results of the non-redundant candidate SNPs in the genomes, the high-quality, paired-end reads were first mapped to the corresponding genomes using SOAPaligner [28,29] (http://soap.genomics.org.cn, version 2.21). Next, the numbers of the most abundant (n1) and second-most abundant (n2) nucleotides at each SNP position in each strain (counted according to the number of reads in each strain supporting this nucleotide) were examined. High quality SNPs were defined as SNPs that satisfied the criteria n1 + n2 ≥ 10 and n1/n2 ≥ 5 and for which the quality score of each mapped base was >20. Raw small InDels shorter than 50 bp were predicted from the regions recognized as gaps in the alignment of syntenic regions. InDels containing more than one mismatch 5 bp upstream or downstream were eliminated. Read validation was then performed on the remaining InDels, and the InDels for which ≥3 query reads mapped to the InDel-removed sequence of the subject were retained.

### Function enrichment of variant genes

We analyzed the relationships between all gene function variations (genes at SV regions or those containing SNPs or InDels) using different gene/protein databases (COG/GO/KEGG). This allowed us to compute the numbers of proteins for each corresponding COG/GO/KEGG term. We determined the GOG/GO/KEGG enrichment terms for the variant genes using a hypergeometric test [34,35] and calculated the P-value. P ≤ 0.05 was considered a significant enrichment of the GOG/GO/KEGG term for the variant gene/protein. We determined the main biological function of differential proteins using function enrichment analysis.

### Phylogenetic analysis

We converted the protein sequence alignment results into multiple amino acid sequences in the CDS regions and aligned multiple sequences in the clustered gene family using the Muscle software (http://www.drive5.com/muscle, v3.8.31). Finally, we generated the gene family tree by analyzing the multiple-sequence alignment results based on Muscle using the Bayes method with the MrBayes software (MrBayes v3.1.2). The dN/dS ratios were calculated using the CODONML software (in paml version 4.4, January 2010) with the GY-HKY model [36].

### Accession numbers

*K. pneumoniae* strains LCT-KP214 and LCT-KP289 genome sequences have been deposited in GenBank under accession numbers AJHE00000000 and ATRO00000000, respectively.

## Abbreviations

SNP: Single nucleotide polymorphism; InDel: Insertion-deletion; NR: Non-redundant protein database; COG: Cluster of orthologous groups of proteins; KEGG: Kyoto encyclopedia of genes and genomes; GO: Gene ontology; ARDB: Antibiotic resistance genes database; RI: Resistance integrin.

## Competing interests

The authors declare that they have no competing interests.

## Authors’ contributions

Conceived and designed the project: CL, YG, LS, WL and WD. Experiments: DC, LS, YG, XF, JW, TL and CF. Bioinformation analysis: WL, YL, YY, TW and LS. Wrote the paper: YG, LS, YL and WL. All authors read and approved the final manuscript.

## Supplementary Material

Additional file 1: Table S1 Enrichment analysis for gene variants in LCT-KP214 and LCT-KP289. Click here for file

Additional file 2: Table S2 Annotation of antibiotic-resistance genes in LCT-KP214 and LCT-KP289. Click here for file

Additional file 3: Figure S1 Flow chart for the selection of *K. pneumoniae* mutants. Click here for file
