# Supplementary figures and images for: Comparative genomic analysis of Klebsiella pneumonia (LCT-KP214) and a mutant strain (LCT-KP289) obtained after spaceflight
Source: BMC Genomics. 2014 Jul 12;15:589. doi: 10.1186/1471-2164-15-589 (PMC4226956; doi:10.1186/1471-2164-15-589)

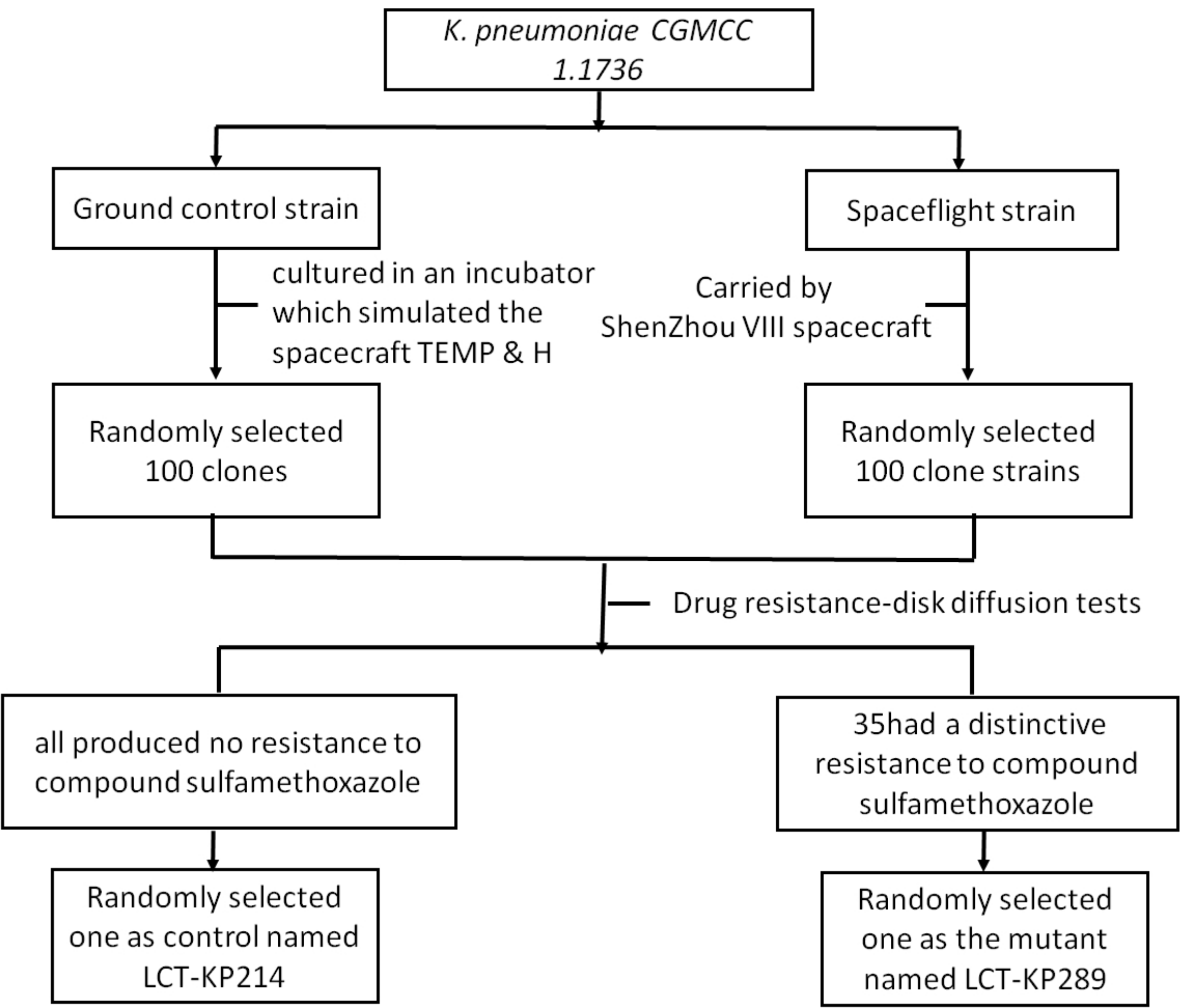

Supplement: Additional file 3: Figure S1 — Flow chart for the selection of K. pneumoniae mutants. [file 1471-2164-15-589-S3.pdf]
